# Examination of risk factors for high Edinburgh postnatal depression scale scores: a retrospective study at a single university hospital in Japan

**DOI:** 10.1186/s40748-024-00176-6

**Published:** 2024-03-04

**Authors:** Tomomi Shiga, Tatsuro Furui, Ken-ichirou Morishige

**Affiliations:** Department of Obstetrics and Gynecology, Gifu University Graduate School of Medicine, 1-1 Yanagido, 501-1194 Gifu-city, Gifu Japan

**Keywords:** Breast feeding, Edinburgh postnatal depression scale, Maternal health, Maternal mental disorders, Neonatal transport, Perinatal mental health, Postpartum depression

## Abstract

**Background:**

Perinatal mental health, such as postpartum depression, is an important issue that can threaten the lives of women and children. It is essential to understand the risk factors in advance and intervene before they can lead to postnatal depression. The risk factors of postpartum depression are reported to vary considerably in Japan. This study aimed to evaluate the risk factors for women with high Edinburgh Postnatal Depression Scale (EPDS) scores and to find women who may need our intervention to prevent postpartum depression.

**Methods:**

This was a retrospective observational study conducted at a single center. At the one-month check-up after birth, the EPDS test was performed in 1625 women who gave birth at our hospital from 2008 to 2016. We evaluated maternal, birth, neonatal and social factors and the breastfeeding status from medical records. Thereafter, we examined the factors that contributed to a high EPDS score.

**Results:**

There were 284 women in the high-score group with an EPDS of ≥ 9, and 1341 women in the low-score group with an EPDS score ≤ of 8. Maternal mental disorders and neonatal transport were significantly associated with high EPDS scores. Conversely, exclusive breastfeeding was significantly associated with the low-score EPDS group.

**Conclusions:**

The principal factor for high EPDS scores was a mental disease. Based on this result, we suggest that early intervention in women at high risk for postpartum depression could prevent serious consequences such as abuse and suicide.

## Background

Perinatal mental health, such as postpartum depression, is an important issue that can threaten the lives of women and children. In Japan, maternal suicide occurs more than twice as often as maternal death due to perinatal complications. Nearly half of the women who committed suicide experienced postpartum depression and other mental disorders [1]. Postpartum depression adversely affects “bonding,” i.e., a mother’s emotional attachment to a child [2, 3]. Women with postpartum depression are more likely to abuse their newborns through actions, such as neglect [4]. Postpartum depression, bonding disorders, and abuse negatively impact the development of the affected children [5, 6], and the adverse effects may be transmitted across generations [7, 8].

The incidence of postpartum depression increased in the country (from 5.0 to 26.32%), and the pooled prevalence was reported to be 14% [9]. Moreover, its prevalence in Japan is 14.3% [10]. A rapid decrease in estrogen and dysregulation of cortisol have been reported to be associated with postpartum depression, but the exact cause remains unknown [11–14]. Various risk factors for postpartum depression were reported, such as depression during pregnancy, being a single mother, young women, grand multipara, low income, low education levels, history of mental disorders, stressful life events (such as bereavement of a loved one, divorce, and unemployment), and lack of social support [15–18]. Japanese studies have also reported risk factors, such as primipara, young women, a perceived lack of family cohesion, lack of social support, current physical illness treatment, psychiatric illness treatment, negative feelings about pregnancy, and combined breast and bottle feeding [19, 20]. However, research on risk factors in Japan is limited to small-scale population-based studies, and the results exhibit significant variability. The interest in postpartum depression has been increasing in recent years, leading to various interventions for prevention. This has further complicated the assessment of risk factors. This means that by conducting more interventions for the high-risk group, there is a possibility that the true risk factors may not be accurately identified. Furthermore, there are not many studies that have evaluated the risk of postpartum depression based on the presence or absence of mental disorders or the details of these.

Our study aims to assess the risk factors in our hospital associated with high EPDS scores before intervention, with the goal of linking it to future activities for the prevention of postpartum depression. Additionally, we explored whether there are differences in risk factors based on the presence of mental disorders and their specifics.

## Methods

### Participants and collected data

This is a retrospective observational study conducted at a single center. We evaluated the data of women who gave birth at our hospital between 2008 and 2016 and who underwent the Edinburgh Postnatal Depression Scale (EPDS) at the first-month of check-up after birth. During this period, we had not yet been able to conduct systematic interventions for the evaluation of risk factors and the prevention of postpartum depression based on them. In principle, all women completed the EPDS questionnaire unless they did not speak Japanese, were undergoing psychiatric treatment due to poor mental status, or were transferred. The EPDS scores at the first-month check-up after birth, maternal factors, birth factors, neonatal factors, breastfeeding status, and social factors were extracted from the medical records. Maternal factors included age at birth, parity, fetus number, pregnancy complication (e.g., hypertensive disorder of pregnancy, gestational diabetes mellitus), underlying maternal disease (e.g., autoimmune disease, heart disease), mental disorder, and maternal transport (i.e., the woman was transferred to our hospital from another hospital due to complications). Birth factors included gestational age at birth and birthing method. Neonatal factors included birth weight, Z-score of the birth weight, hospitalization at the neonatal intensive care unit (NICU), and neonatal transport (i.e., the newborn was transported from our hospital to another hospital due to complications). Social factors included the presence of the partner, welfare, and late visit (i.e., the first visit was after 22 gestational weeks).

### Risk assessment of high EPDS score

The cutoff value for EPDS was set at 8/9, as noted by Okano et al., who demonstrated its highest detection rate for postpartum depression in the Japanese version of EPDS (the sensitivity was 0.75 and the specificity was 0.95) [21]. Women were assigned to a high-score group with an EPDS of ≥ 9 and a low-score group with an EPDS of ≤ 8. The following items were compared between the groups: age at birth, number of young pregnant women (age at birth ≤ 19 years), advanced maternal age (age at birth ≥ 40 years), primipara, multiple pregnancy, pregnancy complications, underlying maternal disease, mental disorder, maternal transport, gestational age at birth, number of preterm birth, urgent caesarean section, birth weight, Z-score of birth weight, low birth weight (< 2500 g), light for date (birth weight < 10th percentile), hospitalization at the NICU, neonatal transport, exclusive breastfeeding, bottle-feeding, no partner, welfare, and late visit. Multivariate analysis was performed using items that revealed significant differences in the univariate analysis, and risk factors that resulted in high EPDS scores were extracted.

### Risk assessment by presence or absence of mental disorders and its details

As a sub-analysis, women were divided into two groups according to the presence of mental disorders, and the backgrounds between the two groups were compared. Risk factors for high EPDS scores in both groups were determined by univariate and multivariate analysis, respectively. In addition, for the group with mental disorders, we investigated whether any factors resulted in a high EPDS score among the differences in disease classification by International Classification of Diseases (ICD)-10, medication, and disease condition. Disease classification by ICD-10 was as follows: F10 -F19, Mental and behavioral disorders due to psychoactive substance use; F20 -F29, Schizophrenia, schizotypal and delusional disorders; F30 -F39, Mood [affective] disorders; F40 -F48, Neurotic, stress-related and somatoform disorders; F50 -F59, Behavioral syndromes associated with physiological disturbances and physical factors; F60 -F69, Disorders of adult personality and behavior; F70 -F79, Mental retardation; F80 -F89, Disorders of psychological development; F90 -F98; Behavioral and emotional disorders with onset usually occurring in childhood and adolescence; F99 -F99, Unspecified mental disorder. Medications included the variables “none”, “single-agent”, “polypharmacy”, or “interruption during pregnancy”. Disease conditions included history only, onset during pregnancy, and postpartum exacerbations.

### Statistical analysis

Statistical analyses were performed as follows. Student’s t-test and chi-square test were used for univariate analysis. For multivariate analysis, binomial logistic regression analysis (forced entry method) was performed using SPSS version 23 (IBM, Armonk, NY, USA). Differences were considered statistically significant at *P* < 0.05.

This study was approved by our ethics review board (29–196). All data were anonymized and participants were allowed to refuse participation by opting out.

## Results

From 2008 to 2016, the total number of births at our hospital was 1861. The EPDS questionnaire was administered to 1625 women, and the implementation rate was 87%. 23% of women did not undergo the EPDS for reasons such as not speaking Japanese, having their one-month checkup at another clinic, or not participating due to poor mental health and already receiving psychiatric treatment. The characteristics of the women are presented in Table 1. Since our hospital is a university hospital with various medical departments, the proportion of pregnancy complications and underlying maternal disease was high. In addition, we have a psychiatric department, so the pregnancy rate with mental disorders was high (5.2%).

**Table 1:** Characteristics of the women

		Average / N	SD / %
Maternal	Age at birth (years)	33.1	± 5.1
	Young pregnant women (≤ 19 years)	22	1.4
	Advanced maternal age (≥ 40 years)	137	8.4
	Primipara (no.)	849	52.2
	Multiple pregnancy (no.)	25	1.5
	Pregnancy complication (no.)	914	56.3
	Underlying maternal disease (no.)	744	45.8
	Mental disorder (no.)	85	5.2
	Maternal transport (no.)	296	18.2
Birth	Gestational age at birth (weeks)	38.0	± 1.9
	Preterm birth (no.)	318	19.6
	Cesarean section (no.)	849	52.3
	Urgent cesarean section (no.)	395	24.3
Neonate	Birth weight (g)	2,826	± 510
	Z-score of birth weight	0.41	± 1.2
	Low birth weight (< 2500 g) (no.)	391	24.1
	Light for date (no.)	117	7.2
	Hospitalization at NICU (no.)	901	55.5
	Neonatal transport (no.)	12	0.7
Lactation	Exclusive breastfeeding (no.)	660	40.6
	Bottle-feeding (no.)	149	9.2
Social	No partner (no.)	31	1.9
	Welfare (no.)	4	0.2
	Late visit (no.)	17	1.0

SD: standard deviation; no.: number; Maternal transport: woman was transferred to our hospital from another hospital due to complications; Light for date: birth weight was below the 10th percentile; NICU: neonatal intensive care unit; Neonatal transport: newborn was transported from our hospital to another hospital due to complications; Late visit: first visit was after 22 weeks

### Risk assessment of high EPDS score

The median EPDS score was 4 (range: 0–28) point. The high-score group with an EPDS of ≥ 9 comprised 284 women, and the low score group with an EPDS of ≤ 8 comprised 1341 women. Table 2 summarizes the characteristics of both groups. Maternal age at birth, gestational age of birth, and birth weight did not differ between the two groups. The Z-score of birth weight was slightly lower in the high-score group. The rate of primipara, underlying maternal disease, mental disorder, light for date, neonatal transport, bottle-feeding, and late visit were higher in the high-score group than in the low-score group. The rate of maternal transport, preterm birth, and exclusive breastfeeding was significantly higher in the low-score group. Binary logistic regression analysis (forced entry method)　was performed using items for which significant differences were found in univariate analysis. Items significantly associated with a high EPDS score of ≥ 9 were considered to indicate maternal mental disorder (*P* = 0.00; odds ratio [OR] 4.86, 95% confidence interval [CI] 2.95–8.02, Standardized Coefficients [B] 1.58) and neonatal transport (*P* = 0.03, OR 3.98, 95% CI 1.14–13.9, B 1.38). Conversely, exclusive breastfeeding was significantly associated with a low EPDS score of ≤ 8 (*P* = 0.01, OR 0.65, 95% CI 0.48–0.87, B -0.44). (Table 3)

**Table 2:** Univariate analysis of the factors contributing to a high EPDS score

		High-score group EPDS ≥ 9 (*N* = 284)		Low-score group EPDS ≤ 8 (*N* = 1341)			
		Average		Average		*P-*value*	*T-*value
Maternal	Age at birth (year)	33.0		33.1		0.688	-0.40
Birth	Gestational age at birth (weeks)	38.1		38.0		0.141	1.47
Neonate	Birth weight (g)	2,809		2,826		0.540	-0.61
	**Z-score of birth weight**	**0.26**		**0.44**		**0.022**	**-2.3**
		N	%	N	%	*P-*value*	Chi-square statistic
Maternal	Young pregnant women (≤ 19 years)	5	1.8	18	1.3	0.588	0.29
	Advanced maternal age (≥ 40 years)	30	10.6	119	8.9	0.370	0.80
	**Primipara**	**167**	**58.8**	**692**	**50.9**	**0.015**	**5.93**
	Multiple pregnancy	4	1.4	21	1.6	0.845	0.04
	Pregnancy complication	151	53.2	763	56.9	0.250	1.32
	**Underlying maternal disease**	**153**	**53.9**	**591**	**44.1**	**0.003**	**9.01**
	**Mental disorder**	**46**	**16.2**	**39**	**2.9**	**0.000**	**83.5**
	**Maternal transport**	**40**	**14.1**	**256**	**19.1**	**0.047**	**3.94**
Birth	**Preterm birth**	**43**	**15.1**	**275**	**20.5**	**0.038**	**4.29**
	Urgent cesarean section	67	23.6	328	24.5	0.757	0.10
Neonate	Low birth weight (< 2500 g)	71	25.0	320	23.9	0.684	0.17
	**Light for date**	**29**	**10.2**	**88**	**6.6**	**0.031**	**4.67**
	Hospitalization at NICU	162	57.0	739	55.1	0.551	0.34
	**Neonatal transport**	**5**	**1.8**	**7**	**0.5**	**0.027**	**4.91**
Lactation	**Exclusive breastfeeding**	**81**	**28.5**	**579**	**43.2**	**0.000**	**21.02**
	**Bottle-feeding**	**51**	**18.0**	**98**	**7.3**	**0.000**	**31.91**
Social	No partner	8	2.8	23	1.7	0.218	1.52
	Welfare	2	0.7	2	0.1	0.086	2.94
	**Late visit**	**7**	**2.5**	**10**	**0.7**	**0.010**	**6.69**

T-test was used to compare mean values (age at birth, gestational age of birth, birth weight, Z-score of birth weight), and chi-square test was used for other items

EPDS: Edinburgh Postnatal Depression Scale; SD: standard deviation; Maternal transport: woman was transferred to our hospital from another hospital due to complications; Light for date: birth weight was below the 10th percentile; NICU: neonatal intensive care unit; Neonatal transport: the newborn was transported from our hospital to another hospital due to complications; Late visit: the first visit was after 22 weeks

* statistically significant (*P* < 0.05); **Bold**: statistically significant

**Table 3:** Binary Logistic Regression Analysis (Forced Entry Method) of the factors that resulted in a high EPDS score

		*P*-value*	Odds Ratio	95% Confidence Interval	Standardized Coefficients
Maternal	Primipara	0.26	1.17	0.90–1.55	0.16
	Underlying maternal disease	0.98	0.10	0.74–1.33	-0.004
	**Mental disorder**	**0.00**	**4.86**	**2.95–8.02**	**1.58**
	Maternal transport	0.48	0.86	0.56–1.32	-0.16
Birth	Preterm birth	0.07	0.68	0.45–1.04	-0.34
	Light for date	0.06	1.58	0.98–2.52	0.45
Neonate	**Neonatal transport**	**0.03**	**3.98**	**1.14–13.9**	**1.38**
Lactation	**Exclusive breastfeeding**	**0.005**	**0.65**	**0.48–0.87**	**-0.44**
	Bottle-feeding	0.06	1.51	0.98–2.32	0.41
Social	Late visit	0.05	2.76	0.99–7.72	1.02

EPDS: Edinburgh Postnatal Depression Scale; Maternal transport: the patient was transferred to our hospital from another hospital due to complications; Light for date: birth weight was below the 10th percentile; Neonatal transport: newborn was transported from our hospital to another hospital due to complications; Late visit: first visit was after 22 weeks

* statistically significant (*P* < 0.05); **Bold**: statistically significant

### Risk assessment by presence or absence of mental disorders and its details

The group with mental disorders had a median EPDS of 10 in 85 women, and the group without mental disorders (i.e., 1540 women) had a median of 5. The group with mental disorders was characterized by more primipara, bottle-feeding, no partner, fewer pregnancy complications, maternal transport, preterm birth, urgent caesarean section, and exclusive breastfeeding (Table 4). This did not indicate that women with mental disorders had a lower risk of pregnancy complications, maternal transport, preterm birth, and urgent caesarean section, but rather was based on the institutional protocol at our hospital for women with high-risk pregnancies.

**Table 4:** Characteristics of the women with mental disorders

		With mental disorder (*N* = 85)		Without mental disorder (*N* = 1540)			
		Average		Average		*P-*value*	*T-*value
	**EPDS score**	**10**		**5**		**0.000**	**10.5**
Maternal	Age at birth (years)	32.2		33.1		0.103	-1.63
Birth	Gestational age of birth (weeks)	38.7		38.0		0.332	3.71
Neonate	Birth weight (g)	2,887		2,822		0.256	1.14
	Z-score of the birth weight	0.24		0.42		0.161	-1.40
		N	%	N	%	*P-*value*	Chi-square statistic
Maternal	Young pregnant women (≤ 19)	2	2.4	21	1.4	0.452	0.57
	Advanced maternal age (≥ 40)	7	8.2	142	9.2	0.759	0.09
	**Primipara**	**59**	**69.4**	**790**	**51.3**	**0.001**	**10.6**
	Multiple pregnancy	3	3.5	22	1.4	0.126	2.35
	**Pregnancy complication**	**32**	**37.6**	**882**	**57.3**	**0.000**	**12.61**
	**Maternal transport**	**5**	**5.9**	**291**	**18.9**	**0.002**	**9.16**
Birth	**Preterm birth**	**8**	**9.4**	**310**	**20.1**	**0.015**	**5.88**
	**Urgent cesarean section**	**11**	**12.9**	**384**	**24.9**	**0.012**	**6.23**
Neonate	Low birth weight (< 2500 g)	22	25.9	369	24.0	0.687	0.16
	Light for date	8	9.4	109	7.1	0.418	0.66
	Hospitalization to NICU	50	58.8	851	55.3	0.520	0.41
	Neonatal transport	0	0.0	12	0.8	0.414	0.67
Lactation	**Exclusive breastfeeding**	**16**	**18.8**	**644**	**41.8**	**0.000**	**18.12**
	**Bottle-feeding**	**37**	**43.5**	**112**	**7.3**	**0.000**	**125.9**
Social	**No partner**	**5**	**5.9**	**26**	**1.7**	**0.006**	**7.57**
	Welfare	0	0	4	0.3	0.638	0.22
	Late visit	1	1.2	16	1.0	0.903	0.02

T-test was used to compare mean values (EPDS score, age at birth, gestational age of birth, birth weight, Z-score of birth weight), and chi-square test was used for other items

EPDS: Edinburgh Postnatal Depression Scale; SD: standard deviation; Maternal transport: patient was transferred to our hospital from another hospital due to complications; Light for date: birth weight was below the 10th percentile; NICU: neonatal intensive care unit; Neonatal transport: newborn was transported from our hospital to another hospital due to complications; Late visit: first visit was after 22 weeks

* statistically significant (*P* < 0.05); **Bold**: statistically significant

Women without mental disease were divided into high and low EPDS score groups, and univariate analysis of risk factors was performed (Table 5). There were 238 women with a high EPDS score of ≥ 9 points and 1302 women in the low-score group with ≤ 8 points. The rates of light for date, neonatal transport, bottle-feeding, and late visit were higher in the high-score group. Only the rate of exclusive breastfeeding was significantly higher in the low-score group. Binary logistic regression analysis (forced entry method) was performed using items for which significant differences were found in univariate analysis. Late visit (*P* = 0.02, OR 3.51, 95% CI 1.26–9.78, B 1.26) was significantly associated with a high EPDS score of ≥ 9 in patients without mental disorder. Conversely, exclusive breastfeeding (*P* = 0.002, OR 0.61, 95% CI 0.45–0.83, B -0.49) was significantly associated with the low-score group with EPDS ≤ 8 (Table 6).

**Table 5:** Chi-square test of the factors contributing to a high EPDS score in the women without mental disorder

		High-score group EPDS ≥ 9 (*N* = 238)		Low-score group EPDS ≤ 8 (*N* = 1302)			
		N	%	N	%	*P-*value*	Chi-square statistic
Maternal	Young pregnant women (≤ 19)	4	1.7	17	1.3	0.646	0.21
	Advanced maternal age (≥ 40)	26	10.9	116	8.9	0.333	0.98
	Primipara	135	56.7	655	50.3	0.069	3.32
	Multiple pregnancy	2	0.8	20	1.5	0.406	0.69
	Pregnancy complication	136	57.1	746	57.3	0.965	0.002
	Maternal underlying disease	107	45.0	552	42.4	0.463	0.54
	Maternal transport	38	16.0	253	19.4	0.209	1.56
Birth	Preterm birth	39	16.4	271	20.8	0.117	2.45
	Urgent cesarean section	61	25.6	323	24.8	0.787	0.07
Neonate	Low birth weight (< 2500 g)	60	25.2	309	23.7	0.623	0.24
	**Light for date**	**24**	**10.1**	**85**	**6.5**	**0.049**	**3.87**
	Hospitalization to NICU	135	56.7	716	55.0	0.622	0.24
	**Neonatal transport**	**5**	**2.1**	**7**	**0.5**	**0.012**	**6.36**
Lactation	**Exclusive breastfeeding**	**73**	**30.7**	**571**	**43.9**	**0.000**	**14.40**
	**Bottle-feeding**	**28**	**11.8**	**84**	**6.5**	**0.004**	**8.45**
Social	No partner	7	2.9	19	1.5	0.103	2.66
	Welfare	2	0.8	2	0.2	0.056	3.66
	**Late visit**	**7**	**2.9**	**9**	**0.7**	**0.002**	**9.91**

EPDS: Edinburgh Postnatal Depression Scale; Maternal transport: woman was transferred to our hospital from another hospital due to complications; Light for date: the birth weight was below the 10th percentile; NICU: neonatal intensive care unit; Neonatal transport: newborn was transported from our hospital to another hospital due to complications; Late visit: first visit was after 22 weeks

* statistically significant (*P* < 0.05); **Bold**: statistically significant

**Table 6:** Binary Logistic Regression Analysis (Forced Entry Method) of the factors contributing to a high EPDS score in the women without mental disorders

		*P*-value*	Odds Ratio	95% Confidence Interval	Standardized Coefficients
Neonate	Light for date	0.10	1.50	0.92–2.44	0.41
	Neonatal transport	0.09	4.91	0.86–9.81	1.07
Lactation	**Exclusive breastfeeding**	**0.002**	**0.61**	**0.45–0.83**	**-0.49**
	Bottle-feeding	0.16	1.41	0.87–2.27	0.34
Social	**Late visit**	**0.02**	**3.51**	**1.26–9.78**	**1.26**

Light for date: birth weight was below the 10th percentile; Neonatal transport: newborn was transported from our hospital to another hospital due to complications; Late visit: first visit was after 22 weeks

* statistically significant (*P* < 0.05); **Bold**: statistically significant

Among the women with mental disorders, 46 were in the EPDS high-score group and 39 in the low-score group. There were no differences between the two groups for any of the items, similar to the other examinations (Table 7). There were no differences between the two groups in disease classification according to ICD-10, medication, and disease condition in the women with mental disorders (Table 8).

**Table 7:** Chi-square test of the factors contributing to a high EPDS score in the women with mental disorders

		High-score group EPDS ≥ 9 (*N* = 46)		Low-score group EPDS ≤ 8 (*N* = 39)		
		N	%	N	%	*P-*value*
Maternal	Young pregnant women (≤ 19)	1	2.2	1	2.6	0.906
	Advanced maternal age (≥ 40)	4	8.7	3	7.7	0.867
	Primipara	32	69.6	27	69.2	0.973
	Multiple pregnancy	2	4.4	1	2.6	0.657
	Pregnancy complication	15	33.3	17	43.6	0.334
	Maternal transport	2	4.3	3	7.7	0.530
Birth	Preterm birth	4	8.7	4	10.3	0.806
	Urgent cesarean section	6	13.0	5	12.8	0.976
Neonate	Low birth weight (< 2500 g)	11	23.9	11	28.2	0.653
	Light for date	5	10.9	3	7.7	0.617
	Hospitalization to NICU	27	60.0	23	59.0	0.924
	Neonatal transport	0	0	0	0	-
Lactation	Exclusive breastfeeding	8	17.4	8	20.5	0.714
	Bottle-feeding	23	50.0	14	35.9	0.191
Social	No partner	1	2.2	4	10.3	0.115
	Welfare	0	0	0	0	-
	Late visit	0	0	1	2.6	0.275

EPDS: Edinburgh Postnatal Depression Scale; Maternal transport: patient was transferred to our hospital from another hospital due to complications; Light for date: birth weight was below the 10th percentile; NICU: neonatal intensive care unit; Neonatal transport: the newborn was transported from our hospital to another hospital due to complications; Late visit: first visit was after 22 weeks

* statistically significant (*P* < 0.05);

**Table 8:** Chi-square test of mental disorder characteristics contributing to a high EPDS score in women with mental disorders

		High-score group EPDS ≥ 9 (*N* = 46)		Low-score group EPDS ≤ 8 (*N* = 39)		
		N	%	N	%	*P-*value*
Disease classification by the ICD-10**	F10-F19	1	2.2	0	0.0	1.000
	F20-F29	6	13.3	8	20.5	0.557
	F30-F39	21	46.7	13	33.3	0.308
	F40-F49	15	33.3	13	33.3	1.000
	F50-F59	6	13.3	4	10.3	0.923
	F60-F69	5	11.1	1	2.6	0.275
	F70-F79	3	6.7	2	5.1	1.000
	F80-F89	0	0	0	0	-
	F90-F98	1	2.2	0	0.0	1.000
Medication	None	17	37.8	16	41.0	0.936
	Single agent	16	35.6	10	25.6	0.457
	Polypharmacy	12	26.7	13	33.3	0.669
	Interruption during pregnancy	9	20.0	5	12.8	0.557
Disease condition	History only	4	8.9	6	15.4	0.563
	Onset during pregnancy	2	4.4	2	5.1	1.000
	Postpartum exacerbation	4	8.9	1	2.6	0.448

EPDS: Edinburgh Postnatal Depression Scale; ICD: International Classification of Diseases; F10-F19: Mental and behavioral disorders due to psychoactive substance use; F20-F29: Schizophrenia, schizotypal and delusional disorders; F30-F39: Mood (affective) disorders; F40-F48: Neurotic, stress-related and somatoform disorders; F50-F59: Behavioral syndromes associated with physiological disturbances and physical factors; F60-F69: Disorders of adult personality and behavior; F70-F79: Mental retardation; F80-F89: Disorders of psychological development; F90-F98: Behavioral and emotional disorders with onset usually occurring in childhood and adolescence; F99-F99: Unspecified mental disorder

* statistically significant (*P* < 0.05)

** There was duplication

## Discussion

The primary findings revealed that risk factors contributing to elevated EPDS scores before intervention included maternal mental disorders and neonatal transport. However, distinct risk factors emerged when patients were categorized based on the presence or absence of mental disorders. In the group without mental disorders, a late visit was the sole significant risk factor for elevated EPDS scores. Conversely, risk factors associated with high EPDS scores were not identified in the group with mental disorders. This implies that the risk of postpartum depression seems to persist irrespective of the type or condition of mental disorders. Exclusive breastfeeding was strongly associated with low EPDS scores overall as well as in the group without mental disorders.

### Maternal mental disorder

Maternal mental disorder is a well-established risk factor for postpartum depression [15, 19, 20]. In our study, EPDS scores tended to be high regardless of the type of mental illness. In some instances, the EPDS level was high even if the woman only had a past medical history and was not currently being managed with any medication. The history of mental illness was mainly self-reported by the woman, whereas some women did not list their past mental disorders on the questionnaire. It is important to recognize that a history of mental illness is a risk factor for postpartum depression and requires a detailed interview at the first visit. Women with mental disorders are more likely to have difficulty parenting because of their characteristics (e.g., clumsy or strength of commitment). In addition, it is known that the employment rate of women with mental disease is low [22], and many were not accompanied by a partner at our hospital. It is expected that these factors will potentiate difficulties in parenting and the life-long relationship. Women with a medical history of mental disease may face various difficulties, including postpartum depression, and thus require early mental, social, and economic intervention.

### Neonatal transfer

According to the Cochrane systematic review by Moore et al. [23], early mother-infant contact effectively establishes mothers’ attachment behavior and mother-child interactions. The effect is shown not only during breastfeeding but also in many attachment behaviors, such as kissing after discharge. Neonatal transport deprives women of the opportunity to touch their infants early after birth, which may impede the mothers’ attachment. In addition, since neonatal transfer means that the infant presents with complications, it is natural that the mother’s anxiety increases.

### Late visit

In Japan, the reasons for late visit have been literature-reviewed and include the following: 1.Economic reasons, 2. Unawareness of pregnancy, 3. No one to consult with, 4. Busy schedule, 5. Aware of pregnancy but neglected it, 6. Lack of knowledge or uncertainty about what to do, 7. Family situations (divorce, domestic violence, infidelity), 8. Undecided about childbirth, 9. Illegal residence, homelessness, 10. Planned abortion [24]. These backgrounds include fetal neglect, unwanted pregnancies, social isolation, etc., making it not hard to imagine the potential risk of postpartum depression.

Late visit in the overall population and neonatal transport in the group without mental disorders did not reach statistical significance in the multivariate analysis, with a wide 95% confidence interval. However, there is a potential for these factors to become significant risk factors with an increase in the number of cases.

### Exclusive breastfeeding

In this study, many women exclusively breastfed in the low EPDS group. However, the causal relationship is unclear; exclusive breastfeeding may provide mental stability, or mental stability may enable exclusive breastfeeding. Oxytocin is a peptide hormone secreted by the posterior pituitary gland. It is secreted in response to cervical and uterine elongation during labor and nipple stimulation from breastfeeding, causing muscle fibers in the mammary gland to contract and promote lactation [25]. Oxytocin reduces cortisol, which is secreted in response to stress, but in women with postpartum depressive symptoms, the oxytocin levels during pregnancy and lactation are low [26, 27] or poorly regulated [28]. There are numerous reports on the early interruption of breastfeeding and postpartum depression [29–33], suggesting the involvement of oxytocin. However, the direct relationship between oxytocin and postpartum depression is unknown. The genotype and the reaction of oxytocin with glucocorticoids may be involved in a complicated manner [34–37]. Breastfeeding may have a positive effect on maternal mental health, but in rare circumstances, considerable stress can be induced by the obsession with exclusive breastfeeding or its failure. Breastfeeding is impaired due to maternal perception of not having a sufficient amount of milk, infant’s failure to thrive, mastitis, etc. [38].. Breastfeeding failure has been reported to be associated with shame and guilt [39]. Therefore, we need to maximize breastfeeding support, but we should not force it.

### Strengths and limitations

The strength of this study lies in the data from a population not subjected to systematic interventions for preventing postpartum depression, allowing for the extraction of true risk factors. Additionally, we examined the differences in risk based on the presence or absence of mental disorders and their specifics.

This study has three limitations. First, this survey was conducted at a university hospital where many women had high-risk pregnancies, and therefore the results may differ from those of the general population. Second, several results with significant differences (mental disorder, neonatal transport, late visit) had wide 95% CI, indicating the need for further investigation with a larger population. Third, we were unable to follow up after the one-month postpartum medical examination. Although the EPDS is a highly accurate tool for predicting postpartum depression, it is important to note that it is a questionnaire-based screening tool and not a diagnostic assessment by a medical professional. It is unknown how many of the 284 people with high EPDS scores developed postnatal depression. It is important for early detection in the women who are more likely to present with perinatal mental health problems and intervene appropriately before severe symptoms develop.

## Conclusion

In our facility, it has been identified that the risk factors for postpartum depression include maternal mental disorders, neonatal transport, and late visits. Additionally, the successful establishment of exclusive breastfeeding has been suggested to have the potential for preventing postpartum depression.

Since 2017, we have been utilizing the findings of this survey as a reference to develop and implement a risk assessment tool, incorporating high EPDS scores. Women identified as high risk for mental health issues undergo interviews, and social support is coordinated. We also provide assistance to women aiming for exclusive breastfeeding to ensure the successful attainment of that goal. Further research is needed to investigate whether proactive interventions based on these risk factors can effectively reduce the incidence of postpartum depression.

## Data Availability

Not applicable.

## Abbreviations

EPDS: Edinburgh Postnatal Depression Scale
NICU: neonatal intensive care unit
ICD: International Classification of Diseases
OR: odds ratio
CI: confidence interval
